# Assessing empathy among caregivers: a cross-sectional study

**DOI:** 10.1192/j.eurpsy.2024.1140

**Published:** 2024-08-27

**Authors:** M. Kahloul, I. Kacem, A. Ghenim, A. Aloui, A. Chouchane, M. Ajmi, W. Naija, M. Maoua, N. Mrizak

**Affiliations:** ^1^Sahloul Academic Hospital, Anesthesia and Intensive Care Department; ^2^Occupational Medicine Department, Farhat Hached Academic hospital, Sousse, Tunisia

## Abstract

**Introduction:**

Empathy plays an important role in everyday human relationships. It is the ability to put oneself in the place of others, to represent what they think and feel. In healthcare settings, several studies have highlighted its positive effects on patients in terms of physical and psychological well-being.

**Objectives:**

Evaluate empathy among caregivers.

**Methods:**

This is a cross-sectional study, conducted over a 1- month -period and enrolling nursing staff working at Farhat Hached Academic hospital. Empathy was assessed using the Jefferson Scale of physician’s empathy (JSPE) scale.

**Results:**

A total of 92 caregivers were enrolled in this study. The average age was 40.41 years with a sex ratio of 0.95. The most represented category was nurses (64.1%) with an average seniority of 17.2 years. The average empathy score was 98.4. Scores above half were reported in 69.5% of cases. The presence of empathy was significantly associated with female gender (p=0.002).

**Conclusions:**

Empathy is a key point in the patient-caregiver relationship. Thus, the nursing staff must be aware of this concept in order to improve the quality of care.

**Disclosure of Interest:**

None Declared

